# Synthetic Biology Tools for Engineering Microbial Cells to Fight Superbugs

**DOI:** 10.3389/fbioe.2022.869206

**Published:** 2022-05-04

**Authors:** Angel León-Buitimea, Francisco de Jesús Balderas-Cisneros, César Rodolfo Garza-Cárdenas, Javier Alberto Garza-Cervantes, José Rubén Morones-Ramírez

**Affiliations:** ^1^ Facultad de Ciencias Químicas, Universidad Autónoma de Nuevo León (UANL), San Nicolás de los Garza, Mexico; ^2^ Centro de Investigación en Biotecnología y Nanotecnología, Facultad de Ciencias Químicas, Parque de Investigación e Innovación Tecnológica, Universidad Autónoma de Nuevo León, Apodaca, Mexico

**Keywords:** synthetic biology, antimicrobial resistance, genetic circuits, antibiotics, phages, whole-cell engineering

## Abstract

With the increase in clinical cases of bacterial infections with multiple antibiotic resistance, the world has entered a health crisis. Overuse, inappropriate prescribing, and lack of innovation of antibiotics have contributed to the surge of microorganisms that can overcome traditional antimicrobial treatments. In 2017, the World Health Organization published a list of pathogenic bacteria, including *Enterococcus faecium, Staphylococcus aureus, Klebsiella pneumoniae, Acinetobacter baumannii*, *Pseudomonas aeruginosa,* and *Escherichia coli* (ESKAPE). These bacteria can adapt to multiple antibiotics and transfer their resistance to other organisms; therefore, studies to find new therapeutic strategies are needed. One of these strategies is synthetic biology geared toward developing new antimicrobial therapies. Synthetic biology is founded on a solid and well-established theoretical framework that provides tools for conceptualizing, designing, and constructing synthetic biological systems. Recent developments in synthetic biology provide tools for engineering synthetic control systems in microbial cells. Applying protein engineering, DNA synthesis, and *in silico* design allows building metabolic pathways and biological circuits to control cellular behavior. Thus, synthetic biology advances have permitted the construction of communication systems between microorganisms where exogenous molecules can control specific population behaviors, induce intracellular signaling, and establish co-dependent networks of microorganisms.

## Introduction

With the increase in clinical cases of bacterial infections with multiple antibiotic resistance, the world has entered a health crisis. Overuse, inappropriate prescribing, and lack of innovation of antibiotics have contributed to the surge of microorganisms that can overcome traditional antimicrobial treatments (Ventola, 2015). In 2017, the World Health Organization published a list of pathogenic bacteria, including *Enterococcus faecium* (*E*. *faecium*), *Staphylococcus aureus* (*S. aureus*), *Klebsiella pneumoniae* (*K. pneumoniae*), *Acinetobacter baumannii* (*A. baumannii*), *Pseudomonas aeruginosa* (*P. aeruginosa*), and *Escherichia coli* (*E. coli*) (ESKAPE) (World Health Organization, 2017). These bacteria have the ability to adapt to multiple antibiotics and transfer their resistance to other organisms; therefore, studies to find new therapeutic strategies are needed. One of these strategies is synthetic biology geared toward developing new antimicrobial therapies.

Synthetic biology is founded on a solid and well-established theoretical framework that provides tools for conceptualizing, designing, and constructing synthetic biological systems (Perrino et al., 2021). Recent developments in synthetic biology provide tools for engineering synthetic control systems in microbial cells. Applying protein engineering, DNA synthesis, and *in silico* design allows building metabolic pathways and biological circuits to control cellular behavior (McCarty and Ledesma-Amaro, 2019). Thus, synthetic biology advances have permitted the construction of communication systems between organisms where exogenous molecules can control specific population behaviors, induce intracellular signaling, and establish co-dependent networks of microorganisms (Hennig et al., 2015; McCarty and Ledesma-Amaro, 2019).

**FIGURE 1:**
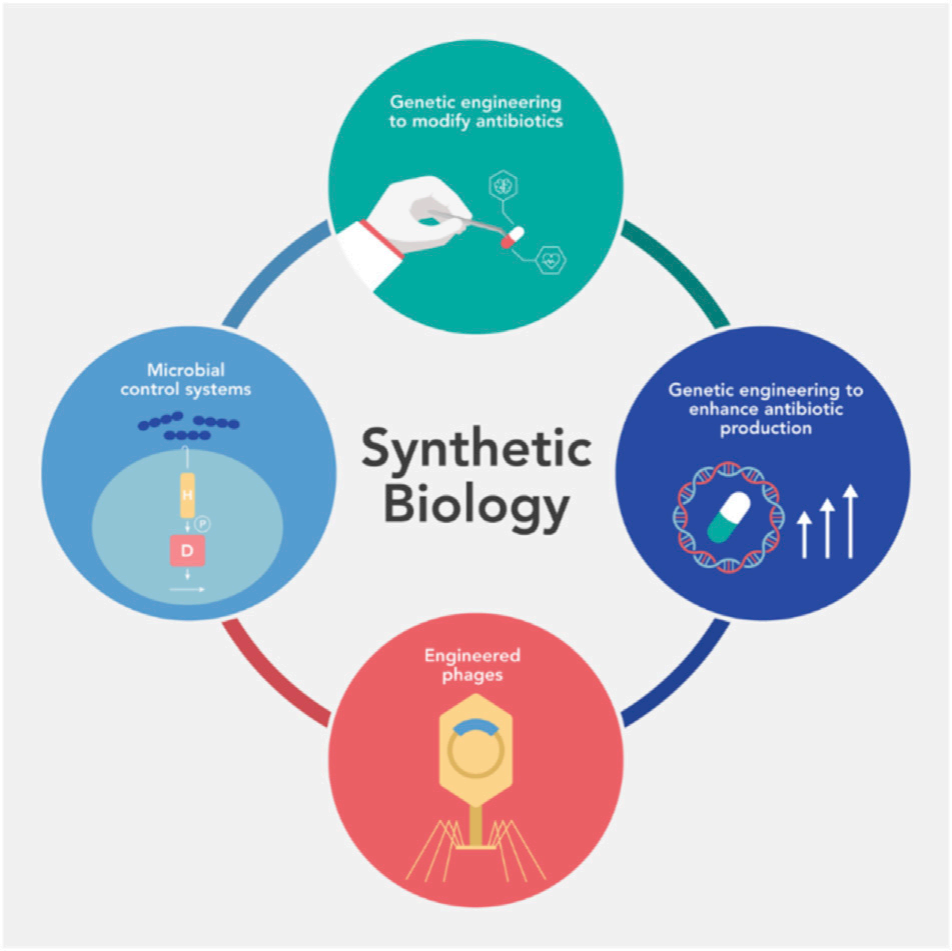
Recent works have demonstrated the impact of synthetic biology and the tools and techniques that the field has developed to allow genetically modifying antibiotics, enhancing antibiotic production, developing engineered phages, and designing microbial control systems.

The applications of synthetic biology systems (artificial circuits and functions within biological systems) include producing biologically based products in agriculture, industry, environmental, and healthcare studies. In this review, we highlight recent works of the impact of synthetic biology on genetic engineering to modify antibiotics and enhance antibiotic production, engineered phages, microbial control systems as an alternative to fight antibiotic resistance, and some other synthetic biology tools to engineer microbial communities (Figure 1).

## Genetic Engineering to Modify Antibiotics

Organisms are naturally capable of producing metabolites with antimicrobial characteristics. Unfortunately, “wild-type” antibiotic producers have poor production and low titers; however, with the unravel of novel biosynthesis mechanisms of that produce antibiotics and the tools provided by synthetic biology, it is possible to engineer the capacities of organisms to over-produce and diversify these metabolites (Breitling and Takano, 2015; El Karoui et al., 2019; Zhang et al., 2020).

One of the most interesting antibiotic synthesis mechanism is the multimodal enzymatic complex presented in biosynthetic cluster genes (BCG). It is composed of non-ribosomal peptide synthetases (NRPS), polyketide synthases (PKS), and combinations. NRPS and PKS are multimodal enzymatic complexes that are responsible for assembling non-ribosomal peptides (NRP) and polyketides, respectively, and giving them an active chemical structure (Martínez-Núñez and López, 2016; Nivina et al., 2019; Hwang et al., 2020). With the tools provided by synthetic biology and applying some engineering to these enzyme complexes, it is possible to attack the problem of low production and limited diversity of antibiotics.

As a first approach to tackle low production and diversity, antimicrobial compounds members of the NRP group have been addressed especially the family of antibiotics known as glycopeptides (Yim et al., 2014). Some authors had focused their efforts on diversifying these antibiotics, such as Yim et al., who used combinations of 13 scaffold-modifying enzymes from 7 GPA BCGs to be introduced into *Streptomyces coelicolor* (*S. coelicolor*). Among these combinations, nine new compounds were reported. Interestingly, eight of those compounds had antimicrobial activity against vancomycin-resistant *Enterococcus faecalis* (*E. faecalis*), exhibiting a MIC between 0.5–4 μg/ml (Yim et al., 2016). One of the promising newcomers in the glycopeptide family is corbomycin. Despite its outstanding clinical performance, natural production with *Streptomyces sp*. has poor performance (Culp et al., 2020). Xu et al. used a glycopeptide antibiotic heterologous expression system (GPAHex) to enhance the expression of genes for corbomicyn synthesis in *S. coelicolor*, obtaining a 19-fold increase in titers using this platform (Xu et al., 2020).

It is also possible to find that combinations between NRPS and PKS modules can give rise to more classes of antibiotics, such as lipopeptides. Daptomycin is a clinically significant lipopeptide antibiotic used primarily against methicillin-resistant *Staphylococcus aureus* (MRSA), naturally produced in *Streptomyces roseosporus* (*S. roseosporus*). Unfortunately, similar to other antibiotic natural producers, “wild-type” *S. roseosporus* has low daptomycin titers (Ye et al., 2014). Recently, Ji et al., using a “top-down” synthetic biology approach, achieved an increase in total lipopeptide production up to ∼2,300%, where up to 40% was daptomycin (Ji et al., 2022).

Not only do combinations between NRPS and PKS give rise to the production of antibiotics, in some microorganisms, these enzyme complexes are joined by other families of enzymes such as fatty acid synthases (FAS). Initially discovered in *Dickeya zeae*, the new family of zeamines is one example of naturally occurring antibiotics resulting from the interaction between NRPS, PKS, and FAS (Wu et al., 2010). Zeamines drew the scientific community’s attention for their potent microbicidal activity against Gram-positive and Gram-negative bacteria. Unfortunately, *Dickeya zeae* has poor production of these antibiotics (Liao et al., 2014). Therefore, different authors, such as Masschelein et al., have studied other natural producers of zeamines, such as *Serratia plymuthica* (*S. plymuthica*) RVH1. Using in-frame deletion of biosynthetic genes, some of the mechanisms involved in synthesizing these antibiotics have been uncovered. This has been an outstanding contribution to synthetic biology for future combinatorial biosynthesis and bioengineering to produce new antibacterial compounds (Masschelein et al., 2013; Masschelein et al., 2015).

In addition to the approaches made by the combination of enzyme complexes, metabolic engineering offers another perspective to improve and diversify the production of antibiotics. Metabolic engineering aims to understand the networks of cellular metabolism and redesign them to improve productive capacities (Olano et al., 2008; Kim et al., 2016; Palazzotto et al., 2019). The efforts provided by metabolic engineering usually focus on redirecting metabolic fluxes, regulating BCGs and enzymes causing bottlenecks.

Discovering the relationships between intermediate metabolites and the biosynthesis of antibiotics have allowed the scientific community to improve yields in antibiotic production. An example of this is the regulation of the metabolite S-Adenosylmethionine (SAM). Although SAM is an essential precursor in methylation processes, this compound is involved in antibiotic biosynthesis in different species (Huh et al., 2004; Wang et al., 2007). Authors, such as Cai et al., manage to increase the yields in bacitracin production in *Bacillus licheniformis* (*B. licheniformis*), analyzing the SAM synthetic pathway. Authors, such as Cai et al., through analyzing the SAM synthetic pathway, have managed to increase the yields in bacitracin production up to 28.97% in *B. licheniformis* with a combination of different synthetic biology techniques such as heterologous expression, deletion, and overexpression of different genes (Cai et al., 2019; Cai et al., 2020).

Up to this point, the described studies have shown that the elucidation of the different mechanisms involved in the biosynthesis of antibiotics and the tools provided by synthetic biology have allowed the redesign of organisms by engineering them to improve their productive capacities and thus contributing to the fight against antimicrobial resistance. Although there are still some critical challenges for the continuous application of synthetic biology strategies to diversify antibiotics, the next few years promise to be rewarding for discovering new antibiotic compounds.

## Genetic Engineering to Enhance Antibiotic Production

Different microorganisms are sources of other compounds used as antimicrobial agents. A variety of genetic material codes those metabolites and accessing this genetic diversity could increase the possibility of finding new or better ways to fight resistant microorganisms. Genetic techniques like gene mutation and the ability to control cellular functions at the genetic level could improve antimicrobial biosynthesis (Miao et al., 2006; Ma et al., 2017). Arafat et al. (2021) observed changes in antibiotic production, exposing *Streptomyces graminofaciens* (*S. graminofaciens*) to UV light. Studying the cellular function of various genes have allowed researchers to produce higher amounts and even better antibiotics. Studies of the gene cluster of the nucleoside antibiotic A201A in *Marinactinospora thermotolerans* (*M*. *thermotolerans*) SCSIO 00652 (Zhu et al., 2012) have led to improving its production; this has also been accomplished through genetic modification for the biosynthesis of amphotericin analogs in *Streptomyces nodosus* (*S. nodosus*) by disruption of *amphDIII* and *amphL* (Byrne et al., 2003). Makitrynskyy developed a manipulation of *AdpA* regions of *Streptomyces ghangensis* (*S. ghangensis*) (Makitrynskyy et al., 2021)*,* giving researchers the understanding of a metabolic pathway to improve a specific antimicrobial biosynthesis. By understanding metabolic pathways and applying different gene engineering techniques, research can improve antimicrobial production through punctual modifications to regulate specific genes, demonstrating that genetic modifications to control biosynthesis pathways could improve antimicrobial production and its activities (Song et al., 2017; Li D. et al., 2021; Li Y.-P. et al., 2021).

Similarly, intended gene modifications can improve antimicrobial production, improve the metabolic flux to precursor availability, and enhance biosynthesis (Meng et al., 2017; Moosmann et al., 2020). These modifications could increase the concentration of antimicrobial precursors, such as (Shomar et al., 2018) expressing the gene cluster for carbapenem in *E. coli* producing antibiotics with a 60-fold increase. Also, modifying metabolic pathways, like carbon flux from the pentose phosphate pathway (PPP) to glycolysis by modifying *zwf1* and *zwf2* on *Streptomyces lividans* (*S. lividans*) (Butler et al., 2002) have led to increased glycolysis intermediates needed for antibiotic production.

A better bio-factory could be achieved by introducing genes in or from a different host taking advantage of its synthesis route (Han et al., 2012; Sakai et al., 2012; Makitrynskyy et al., 2021). Chen *et al.* (Chen et al., 2009) studied the expression of a gene cluster of *Streptomyces cacaoi* (*S. cacaoi*) in *S. lividans* TK24. However, polyoxin production by *S. lividans* TK24 was entirely the polyoxin H derivate due to the lack of genes for hydroxylation/carboxylation, leading to the formation of polyoxin A/F derivates. Eustáquio *et al.* (Eustáquio et al., 2004) expressed the gene cluster to synthesize novobiocin in *S. coelicolor*. Two *S. coelicolor* mutants were obtained by modifying the *novO* gene, an 8′-unsubstituted novobiocin by inactivation of *novO*, and a chlorine atom at C-8′ by expressing the *clo-hal* gene from the clorobiocin gene cluster. Wang *et al.* (Wang et al., 2021) expressed CYP genes of the mushroom *Ganoderma lucidum* in *Saccharomyces cerevisiae* (*S. cerevisiae*) to produce a derivate of 3,28-dihydroxy-lanosta-8,24-dien-26-oic acid. By improving the copy number of two resistance plasmids, the production of this new antibiotic was increased 8.2 folds compared to the control strain. The expression of non-ribosomal peptide synthetases in *Bacillus subtilis* (*B. subtilis*) was studied by Eppelmann *et al.* (Eppelmann et al., 2001) by introducing the bacitracin biosynthesis gene cluster of *B. licheniformis*. *B. subtilis* showed comparable self-resistance to bacitracin due to the gene replacement and increased bacitracin A production due to the high-level expression of the bacitracin synthetase and the higher growth rate compared to *B. licheniformis*. Similarly, Wu *et al.* (Wu et al., 2015) expressed the gene *ram29* of the ramoplanin producer *Actinoplanes* sp. into *Streptomyces fungicidius* (*S. fungicidius*)to produce monomannosylated enduracin derivates.

The main goal of creating an engineered microorganism is the increased production of a specific antimicrobial. Nevertheless, when genetic engineering is focused on manipulating foreign genes in an organism, various reasons could affect its biosynthesis. Modifying genes of a particular biosynthesis or metabolic pathway may be enhanced in production but could decrease its antimicrobial activity. Even in the same genre, the differences between strains give the possibility of obtaining derivates of the intended antimicrobial, which could also modify its action. Thus, knowing how a microorganism’s genetic material helps it survive in the microbiome can help us acquire the tools to manipulate it and use it to our advantage, increasing the possibility of obtaining new and better antibiotics.

## Engineered Phages to Fight Superbugs

Phages are viruses that infect specific bacteria and are the most abundant organisms on Earth (up to 2.5 × 10^8^ phages per mL in water) (Bergh et al., 1989). Phages need bacteria to replicate and survive; thus, naturally, they work as bacteria controllers. Phages were discovered at the beginning of the XX century by Frederick Tort and Felix d’Herelle (Sharma et al., 2017), but antibiotics rapidly replaced phages in treating microorganism infections. However, due to the emergence of multi-antibiotic resistant bacterial pathogens, phages are considered an alternative way to treat multi-resistant bacteria.

Nowadays, researchers are studying single phages, discovering new phages, and designing phage cocktails as therapies to treat multi-resistant bacterial infections. Natural phages could be enough to fight superbugs, just like the works reporting on the administration of phage cocktails to patients infected by a multi-drug resistant *A. baumannii* strain, with results showing complete recovery after phage therapy (Schooley et al., 2017). Nevertheless, synthetic biology can improve or extend phage’s abilities to generate variants with unique properties (Yacoby et al., 2007; Edgar et al., 2012). One of the strategies is to design and build phages with a broad host range (Lin et al., 2012). Phages provide a highly specific target of bacterial strains; thus, cocktails are required to fight infections and reduce the possibility that bacterium acquires resistance to any phage. Yehl et al. (2019) have developed a high-throughput strategy to engineer host-range-determining regions (HRDRs) in T3 phage by site-directed mutagenesis. Inspired by antibody specify engineering, this approach reduces disruptions in tail structure, and they call it “phage bodies”.

Following this strategy could reduce the number of phages in cocktails. Using the monophages approach reduces the preparation and purification efforts and there is less potential for complications derived from using phages cocktails, such as phage purification, phage compatibility, making it easier to use as treatment. In addition, cocktails could make bacteria develop “broad-spectrum” mechanisms of phage resistance, such as capsules that avoid phage binding (Schooley et al., 2017).

Furthermore, Ando et al. (2015) swapped tail fiber genes to allow a genetically *E. coli* to target *Klebsiella* and vice versa. The authors also demonstrated that synthetic phage cocktails with the same scaffold, but different tails selectively remove bacteria from multi-specie communities. Bacterial biofilms are difficult to eradicate since the physical properties of matrix, the physicochemical properties of the exopolysaccharides, and the heterogeneity of the bacterial cells within the biofilm confers them a strong resistance against chemicals (antibiotics, immunological approaches, and phages). Nevertheless, phages genetically engineered could be used to overcome this problem. Chen et al. (2021) constructed an engineered T4 called T4 Rn11 that exhibits antibiofilm activity against *Streptococcus mutants* (*S. mutants*). A reduction in biofilm biomass and formation of microcolonies was achieved using this phage. In a similar work, Pei and Lamas-Samanamud (2014) constructed a T7 phage that produces a lactonase enzyme with broad-range activity for quenching quorum sensing. The modified phage effectively degrades acyl-homoserine lactones (AHLs) from many bacteria. In addition, it inhibited *P. aeruginosa* and *E. coli* biofilm formation.

Reducing biofilm is crucial in the treatment of infection caused by microorganisms in cystic fibrosis disease (Dedrick et al., 2019) or in wound infections, but also it is useful in reducing biofilm in medical devices such as catheters (Fu et al., 2010). These devices are responsible for substantial morbidity and mortality among patients.

As we described above, combining phage’s natural abilities with synthetic biology tools can improve the potential for phages by expanding their range of infection and mediating bacterial signaling or expressing enzymes (Bikard et al., 2014) that help eliminate multi-resistant bacteria.

## Microbial Control Systems

In the last decade, synthetic biology tools have allowed the construction of microbial control systems by engineering whole living cells to act as biosensors and detect and respond to internal and external signals [quorum sensing (QS)] secreted by pathogens (Benítez-Chao et al., 2021; Perrino et al., 2021). Some QS-based phenotypical behaviors in bacteria are sporulation formation, virulence factors related to invasion, bioluminescence, and population control (Benítez-Chao et al., 2021).

In numerous studies, synthetic genetic circuits have been developed to analyze biological systems and guide their design based on QS. A group of researchers genetically modified *E. coli* to detect wild-type *P. aeruginosa* (PAO1), specifically via its QS molecule. Their results demonstrated that engineered *E. coli* sentinels successfully inhibit PAO1 growth by secreting a novel pathogen-specific engineered chimeric bacteriocin (Gupta et al., 2013). Later, a *Lactococcus lactis* (*L. lactis*) to detect *E. faecalis* was designed in another study. *L. lactis* was able to produce and secrete peptides that inhibit enterococcal growth and reduce the viability of enterococci in the surrounding area of *L. lactis*. This engineered system was demonstrated to be effective against multidrug-resistant *E. faecium* strains (Borrero et al., 2015). Holowko et al. (2016) designed and created a synthetic genetic sensing system using nonpathogenic *E. coli* as the host based on CRISPRi technology. They moved proteins used by *Vibrio cholerae* (*V. cholerae*) for QS into *E. coli* and showed high sensitivity to the presence of *V. cholerae* supernatant with tight control of expression of output GFP protein (Holowko et al., 2016). Lubkowicz et al. (2018) developed a probiotic lactic acid bacteria (*Lactobacillus reuteri*) engineered to detect autoinducer peptide-I (AIP-I), a quorum-sensing molecule produced by *Staphylococcus* sp. during pathogenesis. Their results showed that the engineered biosensor could detect AIP-I levels under various strenuous conditions in the *S. aureus* (Lubkowicz et al., 2018). Recently, Wu *et al.* created a novel whole-cell biosensor to detect bacterial pathogens (*P. aeruginosa* and *Burkholderia pseudomallei*) by responding to the relevant QS signal molecules. Results showed that engineered whole-cell biosensors provide rapid and cost-effective detection of waterborne pathogens (Wu et al., 2021).

As described above, quorum sensing is a process in which bacteria communicate with their own species or across species to coordinate cellular behavior. In the last decade, synthetic biology has used the properties of quorum sensing as a tool to build and develop genetic circuits for population control. Thus, scientists seek to design biological systems with predictable behavior. Table 1 displays a summary of the synthetic biology tools to fight antibiotic resistance using genetic engineering to modify antibiotics and enhance antibiotic production, engineered phages, and microbial control systems.

**TABLE 1:** Synthetic biology tools to fight antibiotic resistance using genetic engineering to modify antibiotics and enhance antibiotic production, engineered phages, and microbial control systems.

**Genetic engineering to modify antibiotics**		
Heterologous Expression	Combinations of 13 scaffold-modifying enzymes from 7 GPA BCGs in *Streptomyces coelicolor*	Yim et al. (2016)
	Corbomicyn improvement with glycopeptide antibiotic heterologous expression system GPAHex in *Streptomyces coelicolor*	Xu et al. (2020)
	Application of heterologous expression, deletion, and overexpression to achieve an increase of bacitraicn yield in *Bacillus licheniformis*	Cai et al. (2019); Cai et al. (2020)
Transcriptional optimization of genes	“Top-down” approach to increase lipopeptide production	Ji et al. (2022)
Deletion of genes	Elucidation of the mechanisms involved in synthesizing antibiotics using in-frame deletion of biosynthetic genes	Masschelein et al. (2013); Masschelein et al. (2015)
**Genetic engineering to enhance antibiotic production**		
Mutations	May increase or decrease antimicrobial synthesis depending of the mutation	Miao et al. (2006); Ma et al. (2017); Arafat et al. (2021)
Gene control (elimination, overexpression)	May enhance the biosynthesis of the antimicrobial compound. May require the modification of different genes to increase its production	Zhu et al. (2012)
		Byrne et al. (2003)
		Makitrynskyy et al. (2021)
		Song et al. (2017); Li et al. (2021a); Li et al. (2021b)
Metabolic pathway modifications	May increase the number of metabolic precursors for a certain biosynthesis pathway	Meng et al. (2017); Moosmann et al. (2020)
	Metabolite’s accumulation may overload the biosynthesis pathway	Shomar et al. (2018)
		Butler et al. (2002)
Gene introduction	Gives the possibility of using a different host for better biosynthesis	Han et al. (2012); Sakai et al. (2012); Makitrynskyy et al. (2021)
	The difference between genres and species may redirect the synthesis to different analogs	Chen et al. (2009)
		Eustáquio et al. (2004)
		Wang et al. (2021)
		Eppelmann et al. (2001)
		Wu et al. (2015)
**Engineered phages to fight superbugs**		
Homologous recombination	Deliver genes to replace antibiotic resistance genes into bacteria	(Edgar et al., 2012)
	Replace genes in phages to shift or broad host ranges	(Lin et al., 2012)
DNA sequence-specific antimicrobials	Deliver CRISPR-Cas9 system into cytoplasm to kill bacteria	(Bikard et al., 2014)
Phage-Display	Conjugate antibiotic with phages to enhance bactericidal activity	(Yacoby et al., 2007)
**Microbial control systems**		
Quorum sensing (QS)	*Escherichia coli* to detect wild-type *Pseudomonas aeruginosa*	Gupta et al. (2013)
	Lactococcus lactis to detect *Enterococcus faecalis*	Borrero et al. (2015)
	Biosensing *Vibrio cholerae* with *Escherichia coli*	Holowko et al. (2016)
	*Lactobacillus* reuteri to detect *Staphylococcus* sp	Lubkowicz et al. (2018)
	*Escherichia coli biosensors to detect Pseudomonas aeruginosa and Burkholderia pseudomallei*	Wu et al. (2021)

## Other Synthetic Biology Tools to Engineer Microbial Communities

Synthetic biology uses engineering principles in biological systems in a predictable, controllable, and standardized manner to get new biological insights and create cells with improved abilities. The potential uses of emerging technologies such as the Design-Build-Test-Learn (DBTL) cycle, protein engineering against multidrug-resistant bacteria, and logic gates are described below.

### Design-Build-Test-Learn Cycle

As we previously described, synthetic biology is a recently emerging discipline used to design or redesign biological systems and give them improved or new qualities. One of the SynBio engineering principles used to create new biological systems is the Design-Build-Test-Learn (DBTL) cycle (Whitford et al., 2021). DBTL cycle is an increasingly adopted metabolic engineering framework that helps systematize cellular activities and increase their efficacy and generalizability (Opgenorth et al., 2019). Thus, the design of biological circuits and high-throughput screening (HTS) technologies have begun to speed up modern drug discovery cycles and produce new medicines. Each phase of the DBTL cycle is a fundamental component of the cycle. The Design component identifies the problem and selects the desired pathway and host. The Build component selects, synthesizes, and assembles parts for the incorporation into the host. The Test component validates the engineered constructs and strains for target molecule production, transcripts, proteins, and metabolites. This phase generates a significant amount of data. The Learn component analyzes the Test data, and the learnings are used to create a novel testable hypothesis and incorporate them into the next cycle (Petzold et al., 2015; Carbonell et al., 2018). With the DBTL cycle, researchers can rapidly construct new biological systems. Coupling the DBTL cycle with engineering technologies can deliver solutions in drug discovery and solve global problems such as antimicrobial resistance.

### Protein Engineering Against Multidrug-Resistant Bacteria

Like endolysins (encoded by bacterial viruses), proteins act by disrupting the bacterial cell wall or other proteins that inhibit the expression of genes related to antibiotic resistance (gene regulators) and leading to cell death. Some proteins alter microbial behavior, the bacterial cell wall and interrupt signals and genes confering multi-drug resistance. Based on these kinds of proteins, synthetic biology techniques are helpful for engineering proteins into making them more stable or efficient. Moreover, synthetic proteins can be created and manufactured such is the case of the Briers et al. (2014) group that engineered endolysins to act as artilysins (outer membrane-penetrating endolysins Briers et al., 2014). One drawback is that endolysins do not have activity against Gram-negative bacteria because of the impermeable lipopolysaccharide layer surrounding their cell wall. Still, artilysins are highly bactericidal against Gram-negative pathogens, including *P. aeruginosa* and *A. baumannii*.

Another example is dCas9 which is a “dead” catalytical protein. The system is the same used in CRISPR-Cas9 technology, with the difference that dCas9 can bind to the DNA but not cut the strands. In this way, dCas9 is targeted to suppress gene transcription. Wang and Nicholaou (2017) designed two CRISPR-dCas9 systems to target the *mecA* promoter in MRSA to repress the gene’s transcription (Wang and Nicholaou, 2017). Although these alternatives are not applied to clinical, they are promising approaches to use alone or combined with antibiotics or another technology.

### Logic Gates

An interesting line of research in synthetic biology is that of making microorganisms work as control elements, sending and receiving signals through logic gates. Logic gates are widely used in electronic devices. These devices have electronic circuits that transform an input signal into another output signal or convert two input signals into one output signal (Wang et al., 2011). In synthetic biology, living cells act as control elements; therefore, living cells can be programmed to produce the precisely desired behavior in response to intra or extracellular signals (Morris et al., 2010). Gene regulatory network that cells use to interact and respond to the environmental signals, can be used to program logic circuits to link cellular sensors and actuators. Genetic logic gates rely on direct control of the transcription, activating a promoter. One example is using these genetic elements to program a cell to recognize quorum-sensing signals. Benítez-Chao et al. (2021) designed a whole-cell biosensor to sense and kill MRSA. Using genetic logic gates, they design a genetic circuit where *E. coli* can recognize quorum-sensing molecules from MRSA and triggers the expression of a bacteriocin that kills MRSA.

Genetic circuits can also be designed to disrupt a metabolic pathway in multi-drug resistant bacteria or to create whole-cell biosensors, responding to a molecule produced by pathogen organisms and producing an antimicrobial compound. Whole-cell biosensors are still in research but have a promising future in controlling pathogen bacteria.

## Conclusion

This review presents recent approaches based on synthetic biology as an emerging tool to produce new therapeutic compounds. Synthetic biology provides a wide variety of techniques that aid in the design and construction of microorganisms capable of creating, producing, and enhancing biological functions to use them to tackle antibiotic resistance. Still, there are significant challenges to overcome, like 1) many biological components lack clear description, 2) the construction of biological systems is complicated and can be unpredictable, and 3) bio-circuit test is complicated and time-consuming. Therefore, it is necessary to keep studying and innovating processes before being translated into human medicine.
